# Laterality-Specific Training Improves Mental Rotation Performance in Young Soccer Players

**DOI:** 10.3389/fpsyg.2018.00220

**Published:** 2018-02-27

**Authors:** Stefanie Pietsch, Petra Jansen

**Affiliations:** Institute of Sport Science, University of Regensburg, Regensburg, Germany

**Keywords:** mental rotation, laterality, soccer training, laterality specific training, visual-spatial abilities

## Abstract

This study investigates the influence of specific soccer training with the non-dominant leg on mental rotation performance of 20 adolescent soccer players between 10 and 11 years of age. While the experimental group performed soccer specific tasks only with the non-dominant foot once a week for 10 weeks, the control group absolved the same exercises with the dominant foot for the same period of time. Both groups performed a mental rotation task and shot, dribbling and ball control tests before and after the 10 week intervention. The most relevant result was that the experimental group showed a significantly larger increase in mental rotation ability than the control group.

## Introduction

It is the main goal of this study to investigate the effect of laterality-specific soccer training on cognitive performance in young soccer players. There is a lot of bilateral transfer research, but none of it is concerned with the investigation of the effects of unilateral foot training on spatial performance, especially mental rotation performance where a link between motor and spatial processes is well established (see Voyer and Jansen, 2017).

### Laterality-Specific Motor Training

Not only because of its relation to brain organization and especially hemispheric specialization for spatial and verbal abilities handedness has been an interesting research topic for many years. Far less examined than handedness is the phenomenon of “footedness,” which is considered a purer kind of laterality, because footedness appears to be less influenced by external and social factors than handedness (Gabbard and Hart, 2000). Footedness like handedness can be linked to hemispheric asymmetry and hemispheric specialization, since the motor cortex of each hemisphere controls contralateral limb movements (Bryden, 2000). Measurement of footedness is more difficult than measurement of handedness, since most activities are conducted with both legs involving a mobilization and a stabilization component, while hand activities mostly are scaled one-handed and therefore the dominant hand is considered to be the preferred hand for most daily routine activities like writing (Nicholls et al., 2013). In contrast to this for tests of footedness usually a foot-preference for standard active tasks (e.g., kicking) compared to stabilizing tasks (e.g., one-leg stance) is determined (Stöckel and Carey, 2016). In foot-preference tests the preferential kicking foot seems to hold a similar position like the preferred hand for writing in hand preference tasks (Peters, 1988). 88% of all women and 83% of all men declare their right leg as the “mobile leg” and the left one as stabilizing element and for most subjects handedness and footedness are collateral (Chapman and Chapman, 1987).

In team and combat sports literature often propagates the prevalence of left-handers and left-footed athletes (Grouios, 2004; Loffing and Hagemann, 2012) because of strategic advantages and innate superior abilities that are beneficial for that kind of sports. Moreover the effects of mixed- and left-footedness on motor abilities are consistent with published results on better brain inter-hemispheric communication (Tran and Voracek, 2016). There are many studies concerning the influence of unilateral hand training on the other hand. Most studies concentrate on the impact of a motor training with the non-dominant hand (Porac, 2016). The result of this studies show that right- and left-handers are able to improve the fine motor coordination skills (like writing and finger tapping) of their non-dominant left hand in relatively brief training periods shorter than 1 month (Ackland and Hendrie, 2004; Teixeira and Okazaki, 2007; Walker and Henneberg, 2007; Stöckel and Weigelt, 2012). An EEG study of Lange et al. (2006) indicates a modification of extrinsic coordinates after drawing- training with the non-dominant hand wherefore inter-hemispheric connections transferred via the corpus callosum seem to play an important role.

Concerning the effects of laterality-specific training Healy et al. (1986) as well as Steenhuis and Bryden (1989) exhibited that laterality differences in the range of handedness increase through practicing unilateral motor coordination tasks.

A special result of laterality-specific training is a bilateral transfer whereby the gain of experience of a trained limb has an impact on the untrained contralateral limb. Training conducted with one foot can induce a positive effect on the performance of the untrained limb conducting the same task (Teixeira, 2000). Haaland and Hoff (2003) found a positive effect of non-dominant foot soccer training in 15- to 20-year-old soccer players on both the dominant and the non-dominant foot. According to Kumar and Mandal (2005) bilateral transfer is more distinct from non-preferred side to preferred side and greater with respect to speed, but not to accuracy. Teixeira et al. (2003) examined the influence of bilateral practice on the modification of lateral performance asymmetries in young soccer players and found a reduced asymmetry index for the group which mainly trained with the non-preferred leg. These results indicate that lateral asymmetries arose of previous unilateral training can be modified by bilateral exercise. Therefore especially for young soccer players it seems to be important to enhance motor coordination training with their non-dominant foot.

### Mental Rotation

There is lot of research which shows the relation of motor tasks, particularly motor tasks, which are conducted with hands and different kinds of cognitive activity, especially the influence on mental rotation performance of children and adults (Wohlschläger and Wohlschläger, 1998; Wiedenbauer and Jansen-Osmann, 2008). Besides spatial visualization and spatial perception (Linn and Petersen, 1985) mental rotation, which is defined as the mental representation and rotation of objects (Shepard and Metzler, 1971) is one of the classical tasks for the measurement of visuospatial thinking and is considered to be a prototype for depicting gender differences in spatial imagery (Peters and Battista, 2008). More precisely mental rotation is classified as an intrinsic and dynamic spatial skill (Uttal et al., 2013), which enables the mental visualization and transformation of different classes of objects. In classic mental rotation tasks like Vandenberg and Kuse (1978) usually a target stimulus (cubes, letters, animals, hands) has to be compared with identical or mirror-reversed versions of the upright presented target figure rotated in picture-plane or in depth.

Funk et al. (2005) reason that the processes underlying mental rotation especially the rotation of figures in mind is not an exclusively mental effort, but that it depends on the individual moving body and therefore is interfered with motor processes. A supporting effect of motor gestures on mental rotation performance was found in adults (Chu and Kita, 2011) and children (Ehrlich et al., 2006). A meta-analysis of Uttal et al. (2013) proved stable and transferable improvement of visuo-spatial abilities via special forms of spatial training even if the post-tests were not conducted immediately after finishing the training period. Beside that, the increase of subjects with initially weaker visuo-spatial abilities was larger than for rather skilled subjects.

### Mental Rotation and Motor Training

As mentioned above manual mental rotation training plays an important role in relation to mental rotation. Wexler et al. (1998) as well as Wohlschläger and Wohlschläger (1998) verified the influence of manual motor training on mental rotation performance. In agreement with the assumption of Frick et al. (2005), Wiedenbauer and Jansen-Osmann (2008) proved that for children the success of manual motor training is not only bound to trained object, but that the process of mental rotation itself can be enhanced.

The positive impact of manual motor training on mental rotation performance (Wiedenbauer and Jansen-Osmann, 2008) has been well examined and Voyer and Jansen (2017) revealed an overall advantage of motor experts in spatial tasks. In general athletes show a better mental rotation performance compared to non-athletic subjects (Ozel et al., 2004; Moreau et al., 2012; Pietsch and Jansen, 2012a; Voyer and Jansen, 2017). In detail experts in types of sport which contain mental manipulation like wrestlers and gymnasts have better mental rotation skills than sporting novices or athletes without special requirements in visuo-spatial abilities like runners (Moreau et al., 2011). Jansen and Heil (2010) as well as Jansen et al. (2011) provided first evidence for the positive impact of special motor training through learning to juggle with three balls, while Blüchel et al. (2013) as well as Pietsch et al. (2017) supplied evidence that special motor coordination training on children results in a significantly higher increase in mental rotation abilities compared to a control group with and without physical activity. This fits with the presumption, that the steady learning of new and challenging movements benefits sensor-motor components that are obviously reflected in spatial performance (Pietsch and Jansen, 2012b).

### Mental Rotation and Laterality

Besides the influence of motor expertise and motor coordination training many studies deal with the connection of mental rotation performance and laterality. Generally spatial and verbal abilities are lateralized to one of the two cerebral hemispheres in most human subjects, even if left- and right-handers show no difference in general cognitive performance (Szaflarski et al., 2006). Spatial and non-verbal skills are ascribed to the right hemisphere, while the left hemisphere is prevalent for verbal skills and language (Hugdahl, 2000). Kolb and Whishaw (2015) proved anatomical differences in brain morphology of right- and left-handers, particularly Jang et al. (2017) found larger basal ganglia in non-right-handers compared to right-handers. Basal ganglia, especially the putamen, seems to be involved in motor preparation and motor performance (Alexander and Crutcher, 1990; Marchand et al., 2008; Stocco et al., 2010). Right-handers’ visuo-spatial skills usually are controlled by the right hemisphere (Vogel et al., 2003), while for left-handers no such general preference or even a small left-hemispheric dominance in frontal and parietal lobes was determined (Shimoda et al., 2008). Concerning mental rotation as a special visuo-spatial skill, both hemispheres are involved in the transformation process (Mellet et al., 2014).

Further it seems to be important, that subjects with very well skilled visuo-spatial abilities show no hemispheric preference while solving mental rotation tasks whereas subjects with minor spatial abilities primarily use their right hemisphere (Vogel et al., 2003). Studies with FMRI of Kucian et al. (2007) revealed a more bilateral activation pattern of adults compared to children while solving mental rotation tasks, which seems to lead to a faster and more effective mental rotation performance.

### Mental Rotation and Laterality Specific Motor Training

Summarizing motor training especially motor coordination training which includes a steady learning of new and challenging movements (Pietsch and Jansen, 2012b) improves mental rotation performance and increases hemisphere lateralization (Shimoda et al., 2008). Even bilateral operating methods (Kucian et al., 2007) seem to support mental rotation ability. Further unilateral motor training benefits the development of a greater lateralization of the controlling hemisphere (Steenhuis and Bryden, 1989) and encourages an interhemispheric bilateral transfer (Teixeira, 2000).

Until now, there is no study, which investigated lateralized foot-training and the effect on mental rotation. We hypothesize that young soccer-players which run through a special motor training program for their non-dominant leg show a higher increase of mental rotation performance compared to soccer-players which complete the same training program with their dominant leg.

## Materials and Methods

### Participants

Twenty secondary school-aged children (1 girl, 19 boys, *mean age* = 10.60, *SD* = 0.503), 16 right footers and 4 left footers took part in this study. The children were recruited from a school soccer base camp from a secondary school in Germany and were randomly assigned to experimental (*mean age* = 10.50, *SD* = 0.527) and non-experimental conditions (*mean age* = 10.70, *SD* = 0.483). All participants are members of soccer clubs and had played soccer for four or more years (*M* = 5.00, *SD* = 0.97) and for at least 5 h per week (*M* = 6.15, *SD* = 1.04). Both, experimental (1 girl, 9 boys, one left footer) and control group (10 boys, three left footers) consisted of ten pupils. All participants and their parents gave their written consent for participation. The experiment was conducted according to the ethical guidelines of the Helsinki declaration. The mental rotation test and the ZVT we conducted are similar to the type of tasks the children have to solve during math classes, therefore these tests seem harmless and morally inoffensive to us (detailed information is given below). The training program was conducted by two secondary school teachers which are qualified soccer coaches and worked with the children already for more than half a year. Soccer tasks and soccer tests (please see below) are acknowledged and typical forms of soccer training. Ethical approval for this study was not required in accordance with conditions outlined in guidelines from German Research Society (dfg, Deutsche Forschungsgesellschaft) where research that carries no additional risk beyond daily activities does not require Research Ethics Board Approval. We communicated all considerations necessary to assess the question of ethical legitimacy of the study. We assure that our research approach is in line with national and international human research ethics policies and that we exposed and communicated all considerations necessary to assess the question of ethical legitimacy of the study.

### Material

#### Mental Rotation Test (MRT)

**FIGURE 1:**
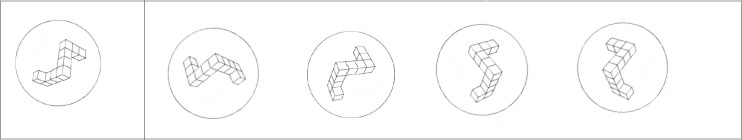
One of 16 items used for testing mental rotation performance (Quaiser-Pohl et al., 2014).

Mental rotation ability was measured by a paper-pencil test with cube figures as stimuli (Quaiser-Pohl et al., 2014), which is based on the MRT of Vandenberg and Kuse (1978). The test consists of 16 items (**Figure 1**), which are displayed on four DIN-A4-sized sheets of paper. Each item contains five figures: on the left-hand side one target figure is presented and on the right-hand side four comparison figures. Two of the four cube figures are picture-plane rotations which are identical to the cube figure on the left, with rotation angles of 45°, 90°, and 135° clockwise/counter clockwise. The other two cube figures are rotated and mirrored versions of the target item on the left. After explaining the concept of mental rotation by two examples the pupils tried to solve as many items as possible in 2 min. The internal consistency (Cronbach’s alpha) of the test is 0.895 (Quaiser-Pohl et al., 2014).

Only if both items that are picture-plane rotation of the target figure were marked was one point given, resulting in a maximal score of 16.

#### Cognitive Speed

The measurement of cognitive speed, cognitive flexibility and executive control, which correlates with mental rotation performance, was conducted by the “Zahlenverbindungstest” ZVT (Oswald and Roth, 1987), a standardized language-independent test procedure. Correlation of the ZVT and standard IQ-tests is *r* = 0.6–0.8 (Vernon, 1993). The internal consistency as well as 6-month test–retest reliability of the ZVT is about 0.90–0.95.

#### Soccer Tests

All soccer tests are based on the testing manual for techno-motoric performance diagnostics of the German soccer association^1^ and were conducted with both feet. For testing ball control pupils had to play as many passes as possible in 30 s against a bouncing wall with a distance of 5 m. The number of correct passes was counted. Dribbling performance was measured by passing through a slalom course while dribbling the ball as fast as possible. Time is measured with light barriers. For the measurement of shot precision the goal was divided in three zones with different strike values, the distance of shot point and goal was added up to seven meters and six shots (three left/three right) were conducted.

#### Exercises

All exercises were designed to train mobilization, motor coordination, and dexterity for especially one foot. Experimental and control group performed the same ball control, dribbling and shot exercises, which were arranged and changed methodically. The experimental group practiced only with their non-dominant foot, while the control group used only the dominant foot. Both groups practiced for 30 min once a week for 10 weeks. For ball control enhancement passing with team mates in different directions and distances was conducted. Dribbling exercises included foot inside and outside dribbling around cones with speed and directional changes. Shot accuracy was practiced by different shooting tasks with static and moving ball.

### Procedure

The testing of the pupils took place during regular base camp time at school in their familiar classroom and gym. Both groups worked through a demographic questionnaire and the ZVT. After that the mental rotation test and on the following day the soccer tests were completed. During training period both groups had one 30-min practice session per week for 10 weeks during their base camp time. After these 10 weeks, the mental rotation test was conducted again 1 day after the post test soccer session. The mental rotation tests were applied in a group session.

### Statistical Analysis

At the beginning the amount of training in years and per week was investigated by two independent *t*-tests for the factor training group. Then, two univariate analysis of variance with the between-subject factor training group (training of the non-dominant foot, dominant foot) and the dependent variables ‘age’ and ‘ZVT’ were conducted. Furthermore, a 2 (group) ^∗^ 2 (time of testing) analysis of variance was computed with the dependent variable ‘number of correct items in the MRT’ and six further 2 (group) ^∗^ 2 (time of testing) analysis of variance with the dependent variables “ball control performance,” “dribbling performance” and “shot performance” for the dominant and the non-dominant foot.

## Results

### Demographic Questionnaire

The two groups did not differ in their amount of training in years, *t*(18) = 1.41, *p* = 0.174 and per week *t*(18) = 1.342, *p* = 0.196.

### MRT

The two groups showed no difference in age*, F*(1,19) = 0.783*, p* = 0.388, $\eta_{p}^{2}$ = 0.042, but in ZVT-performance, *F*(1,19) = 8.517, *p* < 0.01, $\eta_{p}^{2}$ = 0.321. The ZVT value was higher in the control-group (*M* = 2.064, *SD* = 0.28) compared to the Experimental group (*M* = 1.74, *SD* = 0.18).

**FIGURE 2:**
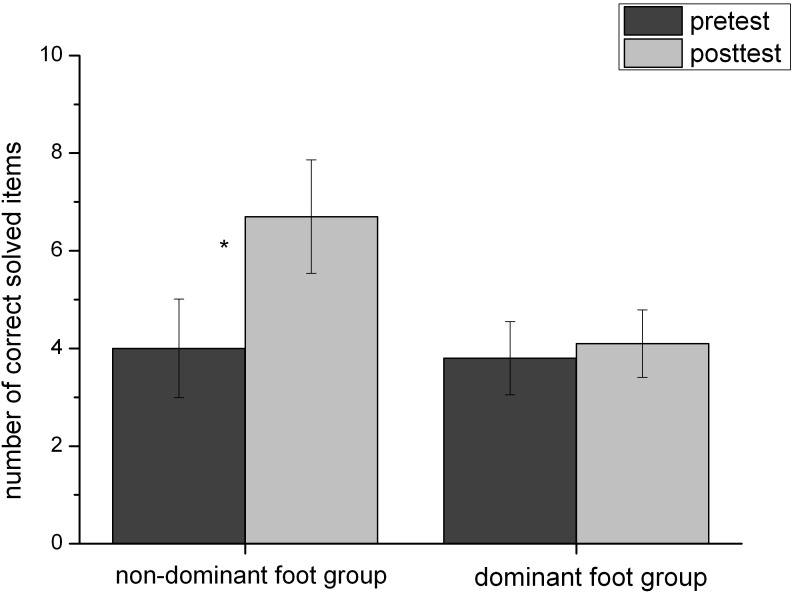
Pre- and post-mental rotation score for non-dominant foot group and dominant foot group (mean and SD).

There was no difference between the groups (EG: *M* = 4.00, *SD* = 3.19; CG: *M* = 3.80, *SD* = 2.39) in mental rotation results in the pre-test*, t*(18) = 0.158*, p* = 0.878. The ANOVA revealed a significant main effect of ‘time’ on mental rotation performance, *F*(1,18) = 12.61, *p* < 0.01, $\eta_{p}^{2}$ = 0.412, performance in the pre-test was lower (*M* = 3.90, *SD* = 2.75) than in the post-test (*M* = 5.40, *SD* = 3.23). The main effect was qualified by a significant interaction between the factors ‘time’ and ‘group,’ *F*(1,18) = 8.07, *p* < 0.05, $\eta_{p}^{2}$ = 0.310 (**Figure 2**). The increase in mental rotation ability was larger (*d* = -0.86) in the non-dominant foot group (post-test *M* = 6.70, *SD* = 3.68) compared to the dominant foot group (post-test *M* = 4.10, *SD* = 2.18). There was even a higher effect-size (*d* = 1.27) concerning the comparison of the difference values between the pre- and post-test for each group (non-dominant foot group: *M* = 2.7, *SD* = 1.49; dominant foot group: *M* = 0.3, *SD* = 2.2). The significant interaction between time and group was also obtained, when the cognitive processing speed was integrated as a co-variate in the analysis of variance mentioned above, *F*(1,17) = 8.392, *p* = 0.01, $\eta_{p}^{2}$ = 0.331.

### Soccer Tests

There were no differences between the groups in all six pre-test measurements (all *p* < 0.01). Because the performance in the soccer test was not the main focus of the study we only present the data without any figures.

#### Shot

The ANOVA displayed a significant main effect of ‘time’ on shot performance for the non-dominant foot, *F*(1,18) = 11.59, *p* < 0.01, $\eta_{p}^{2}$ = 0.392, with lower performance in the pre-test (*M* = 5.25, *SD* = 1.71) than in the post-test (*M* = 6.50, *SD* = 1.53), but not for the dominant foot, *F*(1,18) = 0.04, *n.s*., $\eta_{p}^{2}$ = 0.002. For the non-dominant foot the main effect of time was qualified by a significant interaction between the factors ‘time’ and ‘group,’ *F*(1,18) = 4.17, *p* < 0.01, $\eta_{p}^{2}$ = 0.188. The non-dominant foot group (pre-test: *M* = 4.80, *SD* = 1.54, post-test: *M* = 6.80, *SD* = 1.54) showed a larger increase in shot performance than the dominant foot group (pre-test: *M* = 5.70, *SD* = 1.82, post-test: *M* = 6.20, *SD* = 1.54).

#### Dribbling

For dribbling a significant main effect of ‘time’ on dribbling performance appeared for the non-dominant foot, *F*(1,18) = 25.38, *p* < 0.01, $\eta_{p}^{2}$ = 0.585, with higher performance in the post-test (*M* = 6.60, *SD* = 1.26) compared to the pre-test (*M* = 4.70, *SD* = 1.82). There was also a significant effect for the factor ‘time’ for the dominant foot, *F*(1,18) = 7.89, *p* < 0.05, $\eta_{p}^{2}$ = 0.305. There was no significant interaction between the factors ‘time’ and ‘group’ for the non-dominant foot, *F*(1,18) = 0, *p* = 1, $\eta_{p}^{2}$ = 0.

#### Ball Control

There was a significant main effect of ‘time’ on ball control for the non-dominant foot group, *F*(1,18) = 11.02, *p* < 0.01, $\eta_{p}^{2}$ = 0.380, performance was better in the post-test (*M* = 6.50, *SD* = 1.43) compared to the pre-test (*M* = 5.80, *SD* = 1.60), but not for the dominant foot group, *F*(1,18) = 0.00, *n.s*, $\eta_{p}^{2}$ = 0.001. We found no significant interaction between the factors ‘time’ and ‘group’ for the dominant and non-dominant foot. There were also no relevant effects concerning the training of the dominant foot.

## Discussion

The main result of this study was a significant effect of laterality-specific motor training for the non-dominant foot on mental rotation performance of young soccer players. Enhancement of mental rotation performance was significantly higher when identical exercises were conducted with the non-dominant foot instead of the dominant foot. In contrast to former studies which investigated either the influence of manual motor training (Wexler et al., 1998; Wiedenbauer and Jansen-Osmann, 2008) or specific training in different types of sport (Moreau et al., 2011) in our study we examined the influence of unilateral soccer-specific coordination tasks conducted only with the non-dominant foot. Therefore we excluded conditional impact as well as influences of bilateral forms of training which could enhance the neural communication and a more effective transfer between the hemispheres (Gorynia and Egenter, 2000; Christman and Propper, 2001).

Our results show, that practicing special unilateral soccer exercises which include a steady learning of new and challenging movements for the non-dominant foot improves mental rotation performance. This seems to be caused by the specific motor coordination tasks (Pietsch et al., 2017) as well as by the development of a greater lateralization of the controlling hemisphere (Steenhuis and Bryden, 1989) and an encouraged interhemispheric bilateral transfer (Teixeira, 2000).

Results of the soccer tests showed no significant improvement in ball control and shot performance for the dominant foot neither for the experimental nor for the control group. While the test group practiced only with the non-dominant foot and therefore did not train their dominant foot, the control group performed all exercises with the dominant foot, which is already well trained in basic tasks like ball control and shot performance in young elite soccer players. For this the training program contained only few new challenging tasks for the dominant foot and therefore an improvement of the dominant foot was neither expected for the experimental nor for the control group. In contrast to that we found a significant improvement of shot of the non-dominant foot for the experimental group which practiced only with the non-dominant foot, but no enhancement for ball control performance and dribbling and no upgrade for the non-dominant foot performance of the control group.

According to the model of sequence control of Hikosaka et al. (1999) and Bapi et al. (2000) sequence learning involves two independent coordinate systems with different neural substrates supplying movement production. First a fast developing, effector-independent component which is represented in visual-spatial coordinates and second an effector-dependent slower developing component which is represented in motor coordinates. The fast developing component is for example characterized by sequential target positions and spatial locations of end effectors. Through practicing specific tasks with the non-dominant foot, soccer players acquire a given sequence in visual-spatial and motor coordinates. At the early stages of learning a sequence is coded in visual-spatial coordinates that depend on explicit knowledge, working memory and attention and eventually practice results in a shift to loops associated with motor coordinate processing. For an enhancement in shot performance the development of visual-spatial coordinates is more important than for passing and dribbling the ball wherefore even mainly dynamic movement sequences represented in motor coordinates are crucial. For this a significant enhancement of ball control performance seems to need specific training for a longer practice period. In the optimization processes during practice the development of the sequence structure is thought to proceed in visual-spatial coordinates, while the structure imposed on the elements are thought to occur in motor coordinates. Additionally while performing complex new moves the kinesthetic and vestibular system is trained (Goldstein, 2011), which prepares athletes to use specific cognitive procedures even when there is no special motor task. This was particularly evident for sensor and motoric information (Cherbuin and Brinkman, 2006), which emphasizes the possibility of enhancing visual-spatial abilities through specific motor coordination training. Not only kinesthetic and vestibular information, but also other kinds of information – such as proprioceptive and efferent motor information and their combination – significantly contribute to the development of an effective spatial representation, which in turn leads to improved spatial abilities (Hatzipanayioti et al., 2014; Santoro et al., 2017a,b). The influence of the different kind of information while during movement training should be investigated in more detail in further studies.

### Limitations

This study provides a first insight of the importance of specific training programs with the foot and leg on mental rotation performance. Further studies should comprise larger experimental groups even if a high effect size according to Cohen’s *d* could be demonstrated. With a larger sample size, the amount of right- and left-footers in each group could be balanced. The small sample size could be responsible for the difference in the ZVT-score, independent of time of testing but in favor for the control group. This difference is not explainable, and should be controlled in further studies with a larger sample size. Beside this, the phenomenon of left- and right-footedness should be considered in more detail, because Jang et al. (2017) assumed differences in motor control based on anatomical differences concerning the volume of basal ganglia and differential manifestation of neural communication (Gorynia and Egenter, 2000; Christman and Propper, 2001). Also the developmental perspective should be investigated in more detail. All children were about 10 years old and were trained in soccer for about 5 years. Therefore they already collected more motor experience with both feet compared to untrained children, which could even have reduced the determined effect.

## Conclusion

We found a significant effect of unilateral coordination training with the non-dominant foot on mental rotation performance of young soccer players. The results indicate that it is important to take a close look not only at the specific type of sport when investigating its influence on visuospatial abilities but also on the relevance of laterality specific impact in motor coordination training. Moreover laterality specific training with the non-dominant foot enhances a positive contralateral learning transfer and promotes bilateral competence which is assumed to be an important factor to reach high levels of competitive play especially in team sports like basketball or soccer (Grouios et al., 2002).

## Author Contributions

SP designed the study and wrote the paper. SP and PJ analyzed the data. PJ critically reviewed the paper.

## Conflict of Interest Statement

The authors declare that the research was conducted in the absence of any commercial or financial relationships that could be construed as a potential conflict of interest.

## Footnotes

1 www.bfv.de/cms/docs/dfb_manual_leistungsdiagnostik_low.pdf
